# Research progress on brain network imaging biomarkers of subjective cognitive decline

**DOI:** 10.3389/fnins.2025.1503955

**Published:** 2025-02-13

**Authors:** Han Yingmei, Wang Chaojie, Zhang Yi, Li Yijie, Zhang Heng, Feng Ze, Li Weiqing, Chu Bingyuan, Wang Feng

**Affiliations:** ^1^Graduate School of Heilongjiang University of Chinese Medicine, Harbin, China; ^2^Acupuncture and Moxibustion Massage College, Liaoning University of Traditional Chinese Medicine, Shenyang, Liaoning, China; ^3^Division of CT and MRI, First Affiliated Hospital, Heilongjiang University of Chinese Medicine, Harbin, China

**Keywords:** subjective cognitive decline, cognitive impairment, neuroimaging, brain network, cognitive reserve, review

## Abstract

**Purpose:**

Subjective cognitive decline (SCD) is an early manifestation of the Alzheimer’s disease (AD) continuum, and accurately diagnosing SCD to differentiate it from neurotypical aging in older adults is a common challenge for researchers.

**Methods:**

This review examines and summarizes relevant studies regarding the neuroimaging of the AD continuum, and comprehensively summarizes and outlines the SCD clinical features characterizing along with the corresponding neuroimaging changes involving structural, functional, and metabolic networks.

**Results:**

The clinical characteristics of SCD include a subjective decline in self-perceived cognitive function, and there are significant imaging changes, such as reductions in gray matter volume in certain brain regions, abnormalities in the integrity of white matter tracts and diffusion metrics, alterations in functional connectivity between different sub-networks or within networks, as well as abnormalities in brain metabolic networks and cerebral blood flow perfusion.

**Conclusion:**

The 147 referenced studies in this paper indicate that exploring the structural, functional, and metabolic network changes in the brain related to SCD through neuroimaging aims to enhance the goals and mission of brain science development programs: “Understanding the Brain,” “Protecting the Brain,” and “Creating the Brain,” thereby strengthening researchers’ investigation into the mechanisms of brain function. Early diagnosis of SCD, along with prompt intervention, can reduce the incidence of AD spectrum while improving patients’ quality of life, even integrating numerous scientific research achievements into unified and established standards and applying them in clinical practice by doctors, thus all encouraging researchers to further investigate SCD issues in older adults.

## Graphical abstract

SCD, Subjective Cognitive Decline; ML, Machine Learning; DL: Deep Learning; Aβ, Amyloid-beta protein; Tau, Tau protein. Note: By FigDraw.

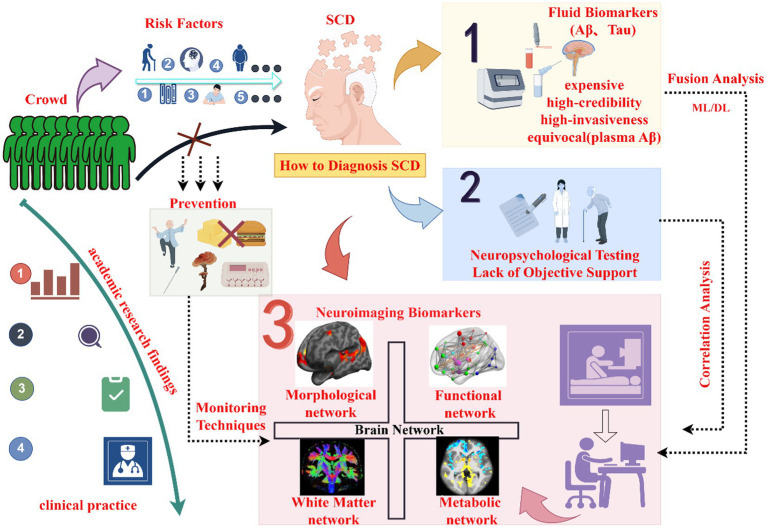

## 1 Introduction

Alzheimer’s Disease (AD) is recognized as the predominant branch of neurodegenerative disorders worldwide. The World Health Organization (WHO) and Alzheimer’s Disease International (ADI) estimating a rise to 139 million by 2050. AD constitutes nearly 65% of dementia cases, with approximately one-fifth of AD patients originating from China, and incidence rates continue to escalate. The detection of AD has increased in part due to better detection tools and increase in lifespan (Heuer et al., 2024). The primary clinical manifestations of AD include cognitive decline accompanied by emotional regulation abnormalities, with clinical treatment efficacy being less optimistic (Singh et al., 2024; Pless et al., 2023).

Research indicates that AD represents a continuum, characterized by the progressive emergence and exacerbation of clinical symptoms following the appearance of neuropathological changes in asymptomatic individuals due to increased pathological burden. Currently, researchers are gradually shifting their focus toward the preclinical stages of AD, namely subjective cognitive decline (SCD; Jessen et al., 2020), which is a typical disorder in the preclinical AD. How to accurately diagnose SCD and adopt preventive measures to halt or delay the further progression of the disease has become a key concern. Meanwhile, converting research findings into clinical practice and providing clear guidance for medical staff regarding the diagnosis, prevention, and treatment of SCD, so as to benefit scholars, has become the common goal among researchers in this field.

In the study of SCD, the AD continuum become a comprehensive term for the evolutionary progression from the absence of cognitive symptoms to severe Alzheimer’s manifestations (Sperling et al., 2011; Albert et al., 2011; McKhann et al., 2011). The AD continuum is divided into SCD, mild cognitive impairment (MCI), and clinical AD. SCD is characterized by neurotypical results in objective tests but self-reported cognitive decline. Complaints such as memory problems, mental slowness, and difficulty in concentrating. These symptoms are often regarded as neurotypical manifestations of aging. In fact, pathological changes have already occurred in the brain, such as higher levels of amyloid-beta (Aβ) deposition than those in neurotypical individuals and abnormal lymphatic functions (Li Y. et al., 2024), exhibiting similar patterns to the observed AD plasma biomarkers and imaging changes (Xu et al., 2023). MCI is defined as having mild memory or other cognitive issues that exceed the normal levels expected for an individual’s age, education, and cultural background, but daily living functions are not significantly impacted. There are no obvious psychopathological symptoms, and the criteria for dementia are not met (Anand and Schoo, 2024). When the stage of AD is reached, research into treatment plans that alleviate and improve symptoms becomes a challenge, and clinical trials often end in failure. Therefore, early diagnosis of AD is crucial for advancing treatment, as it is widely recognized as the key to effective intervention and a significant difficulty in disease prevention and control.

Currently, The National Institute on Aging (NIA) and the Alzheimer’s Association (AA) workgroup advocate for defining the diagnosis of AD continuum should depend on the presence and nature of the biomarker findings and not solely rely on clinical syndromes, such as neuropsychological tests (Albert et al., 2011). Although the cognitive conditions of patients can be grasped by assessing the scores of neuropsychological scales during the stage of AD, conventional cognitive function assessment scales possess limited superiority in detecting SCD. Furthermore, the neuropsychological features of different subtypes of SCD are characterized by heterogeneity. Clinical diagnosis and neuropsychological approaches are incapable of providing objective support, whereas biomarkers are able to do so. This perspective had already surfaced in the literature as early as 2015 (Lista et al., 2015), thereby propelling the research on SCD biomarkers to become the mainstream trend in contemporary research.

According to research findings, cerebrospinal fluid *β*-amyloid and tau proteins are typical fluid biomarkers of the AD continuum (Lantero-Rodriguez et al., 2024), but their detection methods suffer from high invasiveness and costliness. Blood-based biomarkers represent another type of fluid marker, showing good diagnostic performance in detecting pathological changes in cognitively unimpaired individuals along the AD continuum (Lehmann et al., 2023; Li J. et al., 2024). Recent studies have found that plasma neurofilament light chain has reliability in predicting the conversion of SCD, early detection of AD, and the progression of cognitive decline (Mazzeo et al., 2024). However, further research is needed to determine whether these biomarkers truly represent central changes, considering their low levels in peripheral blood and susceptibility to clearance by the human body’s clearing systems. Besides fluid biomarkers, imaging biomarkers are also utilized in AD biology (Ebenau et al., 2022; Wang H. et al., 2023). With the rapid development and widespread adoption of medical imaging technologies, imaging biomarker detection has become one of the main approaches in analyzing AD biological markers (Shahidi et al., 2023), offering advantages such as repeatability, non-radiative, and high spatial resolution during data acquisition. Thus, imaging research has become a hot spot in the field of AD continuum research.

Brain network imaging research provides a multi-dimensional perspective on the brain work effect mechanism of diseases. It evaluates the separation and integration functions of the brain network through the average cluster coefficient and the average characteristic path length (van Diessen et al., 2013). A large body of literature indicates that structural, functional, and metabolic disruptions, as well as damage to topological properties, have already occurred in brain networks during the preclinical stage of AD. However, given the existence of compensatory mechanisms within brain networks, clinical symptoms may remain undetected or manifest as relatively mild conditions (Skouras et al., 2019). Additionally, some researchers believe that AD-related cognitive impairments are the result of brain network damage (Zhang Z. et al., 2023). Therefore, this review, based on neuroimaging research, summarizes the disease evolution process of SCD, exploring the potential connection between clinical characteristics and imaging brain network, and provide an overview of the role of imaging biomarkers in SCD. Summarize the achievements of the existing literature on detecting SCD and the challenges we failed to solve.

## 2 SCD neuroimaging biomarkers

SCD refers to the continuous decline of cognitive ability and self-experience unrelated to acute events. However, cognitive assessments reveal no objective memory problems at this stage (Munro et al., 2023). Studies have found that patients in this stage exhibit metacognitive abnormalities that precede cognitive impairment. Subsequently, the latest studies have demonstrated that metacognition is the result of the role played by the underlying neuropsychology of SCD, and it is a kind of cognition in patients’ self-assessment (Cappa et al., 2024). Additionally, throughout the disease progression along the AD continuum, a negative correlation is observed between metamemory scores and cognitive function, supporting the concept of the AD continuum (Li et al., 2022). Therefore, SCD is a critical point in the transition from normal cognition to clinical AD, as well as a key point for intervention and treatment. Studies have found that SCD is associated with factors such as advanced age, female, anemia, deficiency in physical exercise, solitary living, mild symptoms of anxiety, years of education received, cognitive reserve as well as genetic factors, which precisely provide entry points for SCD intervention and treatment.

In recent years, SCD has become a research hotspot among many researchers. For the diagnosis of SCD, how to distinguish it from neurotypical people and MCI is the main research difficulties at present. Research shows that the neuroimaging studies have provided an advanced perspective for exploring the brain mechanisms underlying SCD, and provided a reliable theoretical basis for the accurate diagnosis of SCD disease.

### 2.1 Gray matter volume and white matter integrity abnormal

In neurotypical individuals, the brain structure undergoes a relatively stable and gradual decrease in average volume with age, encompassing both gray matter (GM) and white matter (WM), albeit with minor variances (Roe et al., 2024). Specifically, the frontal and parietal lobes are more substantially impacted by age, whereas the temporal and occipital lobes are less affected. Intriguingly, the globus pallidus exhibits a positive association with age (Bagarinao et al., 2022). However, in the context of a substantial time span, if the brain structure presents an atrophy rate deviating from the normal and resembling that of AD, such an individual is regarded as an older adults at a heightened risk of progressing to AD.

Studies have made it clear that there are abnormalities in the anatomical structure of the brain areas such as globus pallidus, hippocampus, amygdala, and nucleus accumbens in the MCI stage (Fu et al., 2021). However, there are different statements about the volume change of the SCD brain region. Some studies measured by voxel-based morphometry (VBM) analysis methods found that, compared with neurotypical people, there was no significant change in the GM volume of SCD (Fu et al., 2021). In the previous structural studies on SCD, Chen S. et al. (2020) conducted a meta-analysis on the existing studies based on VBM analysis and discovered that the right lingual gyrus, right cuneus, and right medial frontal gyrus were the main brain regions with atrophy in SCD. Moreover, some researchers have verified, by employing both VBM and region of interest (ROI) analysis methods, that the cortical volume in memory-related brain regions among patients with SCD has exhibited atrophy. Specifically, in regions such as the frontal lobe, occipital lobe as well as the medial temporal lobe, and even in the WM regions including the cingulum, uncinate fasciculus, inferior fronto-occipital fasciculus and anterior thalamic radiation, their volumes have also decreased significantly (Riverol et al., 2024). The inconsistency between these two claims might be attributed to the collection of imaging data from diverse samples of patients with SCD, or it could be associated with the heterogeneity of SCD. Alternatively, it may also be linked to the varying research methodologies employed by investigators. Pini and colleagues were aware of the heterogeneity in the structural alterations of the SCD brain. They gathered imaging data of SCD patients from both community and clinical samples and discovered that there existed discrepancies in the brain atrophy patterns of SCD derived from different samples (Pini and Wennberg, 2021). Consequently, this emphasizes the indispensability of structural research.

In fact, compared with GM volume measurement, white matter (WM) are more sensitive in demonstrating the changes in brain microstructure of SCD.WM is composed of thousands of nerve fibers in the human brain, which connect neurons in different brain intervals to form a complex and stable white matter network, which is the structural basis of neuronal activity and information transmission. The integrity of WM can provide early biomarkers for SCD patients who have not shown any cognitive decline indicators or GM volume defects obtained from objective measurements (Fogel et al., 2021), thus contributing to the early prevention of clinical AD. Using regional spatial statistical and ROI analysis methods, it was discovered that the white matter integrity in the left superior frontal gyrus, right anterior cingulate gyrus, and left lingual gyrus of SCD patients was abnormal before objective cognitive impairment emerged. Thus, it is speculated that the degradation of specific white matter beams is the basis for the decline of cognitive ability of SCD patients (Chao et al., 2022).

For individuals with SCD, when cerebral small vessel disease occurs, the risk factor for progressing to vascular cognitive impairment also increases (Liu et al., 2024). White matter hyperintensities (WMH) are a valid indicator of the occurrence of cerebral small vessel disease. Some researchers collected MRI images of cognitively unimpaired (CU) individuals, those with SCD, and those with MCI from the Alzheimer’s Disease Neuroimaging Initiative (ADNI) database and observed that, compared with CU, the WMH in the temporal, occipital, and frontal lobes of those with SCD were significantly increased, while those with MCI had a wider range of white matter hyperintensity volume in the temporal, occipital, and frontal lobes mentioned above (Calcetas et al., 2022). It can be seen from this that SCD and MCI have different evolutionary outcomes under different circumstances.

Some research literature has demonstrated that in the cerebral cortex of patients afflicted with subjective cognitive decline (SCD), the volumes of specific white matter tracts associated with cognitive functionality, such as the anterior thalamic radiation, superior and inferior longitudinal fasciculi, and the uncinate fasciculus of the corpus callosum, are not merely reduced. Additionally, their diffusion tensor imaging metrics display pronounced aberrations (Wang et al., 2021). DTI is a special sequence based on magnetic resonance imaging technology, which has advantages in evaluating the microstructure of the brain white matter, detecting the diffusion characteristics of brain nerve fiber bundles, revealing the running and integrity of the fiber bundle, and quantifying the microstructural characteristics of the white matter pathway, thus providing researchers with morphological information of brain nerve fibers in SCD patients. Archer et al. relying on the high-resolution white matter fiber tract template of the human brain and applying the ROI analysis technique, quantified the diffusion parameters in regions such as the cingulate gyrus, uncinate fasciculus, superior longitudinal fasciculus, inferior longitudinal fasciculus and fronto-parietal fasciculus of patients with SCD. The results demonstrated that, in terms of evaluating the cognitive decline of SCD, the diffusion indices of the aforementioned regions were more sensitive than the medial temporal lobe volume atrophy and cerebrospinal fluid Aβ. Meanwhile, the study indicated that early demyelination of white matter is an independent pathogenic risk factor for SCD (Archer et al., 2021), thereby validating the above conclusion.

### 2.2 Structural damage to the brain network

The brain network structures of different SCD individuals exhibit differences. The disruption of white matter integrity mentioned above is the structural basis for the formation of brain networks, and the research on the structural damage of brain networks can evaluate the correlation with cognitive function changes from a more comprehensive perspective. It mainly includes morphological network and white matter network.

Based on the morphological network constructed by MRI conventional sequence T1WI combined with graph theory analysis, it can characterize morphological indicators such as cerebral cortex thickness, volume, surface area, sulcus depth, etc., and obtain the morphological relationship of the cerebral cortex by calculating the average Pierson correlation coefficient. The characteristics of global brain morphological network integration and segregation in patients with SCD are significantly different from those in healthy individuals. The results of different studies tend to be consistent in this regard. However, the research findings regarding the Rich-Club of SCD morphological networks are diverse. The Rich-club refers to the phenomenon in the brain network where nodes with high degree values are closely connected, similar to the social concept that “the rich interact with the rich and the poor with the poor,” which is of great significance for information integration and network stability of the brain network. Fu et al. (2022) believe that Rich-club is retained, while Peng et al. (2022) believe that Rich-club has tissue disorders, and its unique disorder mode has become a new insight into the potential mechanism of SCD.

Based on the morphological network analysis, at the global level, the low global efficiency and the shorter normalized characteristic path length constitute the network topological features of SCD (Ding et al., 2023). At the local level, the node attribute indices of the frontal, limbic and parietal regions in the default mode network (DMN) and frontoparietal task control (FTC) network possess strong discriminatory ability in the experiments differentiating neurotypical individuals from SCD patients, and the SCD network shows a tendency of modularity and enhanced local efficiency (Xu et al., 2022). Subsequently, in the research evaluating the association between morphological abnormalities and disease severity, it was also found that the morphological connection between the somatic motor network and the ventral attention network in SCD patients was significantly reduced compared with that in neurotypical individuals, and the morphological network of MCI patients was even more abnormal (Chen et al., 2024).

The white matter network constructed based on the analysis of DTI sequence combined with graph theory, while detecting the white matter integrity of the brain microstructure, it analyses the large-scale network topological properties and the brain structure connection mode of the human brain system. The white matter network acquired based on support vector machines can accurately identify SCD and MCI among the population (Huang et al., 2021), and can also be used to distinguish different types of cognitive impairment (Zhang H. Q. et al., 2023). Among many types of cognitive impairment, the trend of white matter network destruction of SCD is the least obvious. Changes in topological metrics are mainly manifested the decrease in global efficiency, local efficiency, average clustering coefficient, and increase in characteristic path length (Shu et al., 2018). In addition, unique brain network changes were found in the brain regions of SCD, such as the superior parietal gyrus, angular gyrus, precuneus, posterior cingulum, putamen, precentral gyrus, postcentral gyrus, and paracentral lobule and other brain regions, which is consistent with the characteristics of MCI and AD networks (Kim et al., 2019). Similarly, the change of WM network topology metrics is also a gradual process in the evolution of AD continuum (Tao et al., 2021).

Thus, it can be seen that the research on the morphological network and white matter network of SCD has revealed their characteristic differences, laying a foundation for further exploring the network dynamic changes during the progression of SCD to more severe cognitive impairments.

### 2.3 Abnormal neuronal activity

During the aging process of healthy adults, not only does the brain structure exhibit a regular atrophy pattern, but the neuronal activities in specific brain regions also change. For example, the activation of the frontal lobe function and the parietal lobe activity decrease (Li et al., 2013). However, in the research on SCD, it has been found that the brain function and neurophysiological activities of SCD patients will show changes prior to the atrophy of the brain structure (Parker et al., 2020). The essence of the change in brain function is the abnormal activity of neurons. For example, the mismatch negativity (MMN), an index obtained through magnetoencephalography recording, can reflect the integrity of the human sensory memory function. Research has shown that magnetic source mismatch negativity occurs in the left inferior parietal lobule and right inferior frontal gyrus of SCD patients and in the inferior parietal lobule of MCI patients (Cheng et al., 2021; Chen P. Y. et al., 2021). Although the subjects of these two studies were not from the same group of patients, their research results exhibited similarities. This further validates that changes in neuronal activity can serve as a research direction for the effect mechanism of the AD continuum. Remarkably, in the latest research, when using SVM for MRI feature classification analysis, it was discovered that the aforementioned brain regions also have atrophy in volume and morphological changes have taken place (Song et al., 2024). This research can be regarded as a significant supplement to the magnetoencephalography research results of SCD and is worthy of further in-depth exploration by researchers.

Spontaneous brain activity is considered to be a stable imaging biomarker of cognitive impairment, which can indirectly reflect functional imaging indicators such as amplitude of low-frequency fiuctuation (ALFF), regional homogeneity (ReHo), functional connectivity (FC) and degree centrality (DC) of the human brain. Some research has utilized the monitoring of neuronal activity complexity to disclose the subtle differences between healthy individuals and SCD patients. It was found that the neuronal activity complexity in the superior temporal gyrus, the inferior parietal lobule, the postcentral gyrus, and the insular gyrus of SCD was decreased (Ni et al., 2021). It is thus speculated that such alternating complexity activities correspond to the individual’s behavioral and cognitive performance. However, researchers attributed the maintenance of normal cognition in SCD individuals to compensatory activities, such as those in the frontal and parietal regions. Nevertheless, considering various factors, the research by Kerbs et al. did not provide conclusive evidence (Krebs et al., 2023).

Brain regions such as the prefrontal lobe, occipital lobe, left angular gyrus and temporal lobe are relatively sensitive to cognitive ability status (Gao Y. et al., 2023). Early research found that the functional connectivity of bilateral frontal poles, caudate lobes, angular gyrus and lingual gyrus in SCD patients was decreased, which provided a certain basis for the imaging assessment of SCD cognitive status (Parker et al., 2020). However, the latest research shows that the left dorsal prefrontal lobe has a relatively strong correlation with speech assessment of cognitive function, and it was detected that the ALFF, ReHo and DC of the left dorsal prefrontal lobe and precuneus in SCD patients were both significantly decreased (Li X. Y. et al., 2024). It can be seen that with the continuous development of the SCD research field, the existing research consensus is also constantly being updated.

### 2.4 Abnormal functional segregation and integration of brain networks

The brain switches functions among networks in a non-completely random manner. Based on functional attributes, brain networks can be divided into two organizational types: one is the brain network corresponding to various basic functions, and the other is related to advanced cognitive functions (Vidaurre et al., 2017). The neural activity of multiple local nodes is assembled into different brain networks according to different functions or spatial anatomical locations. Through the analysis of brain functional connectomes, the separation of brain functional networks and the abnormal connection within or between networks can provide a variety of information for the diagnosis of SCD. Existing research literature has found that SCD are unable to flexibly and dynamically up-regulate and down-regulate brain network activities, which means that the brain’s dynamics is reduced, and there are also differences in the activation levels of different brain networks. Such changes in brain dynamic characteristics with spatial distribution features are helpful for accurately identifying SCD individuals from the neurotypical population (Chen Q. et al., 2023).

Using resting-state functional magnetic resonance imaging (rs-fMRI) technology, it has been detected that the brain dynamic functional networks during the SCD stage undergo reconstruction, showing imbalanced cortical network reconstruction, as well as disorganization, disruption, and hyperconnectivity of networks. This involves multiple networks, including the DMN, frontal executive network, parietal network, visual network, auditory network, fronto-parietal network (FPN), and limbic network (LN). In addition, a relatively less common network has been identified: the self-referential network (SRN), which interacts with memory-related networks. During the SCD stage, it exhibits a state of hyperconnectivity, whereas in the MCI and AD stages, it shows low connectivity (Chen S. et al., 2021).

The disruption of functional connectivity in the parietal network (Lazarou et al., 2020), visual network (Chen et al., 2021a), DMN, and frontal executive network (Serra et al., 2023) serves as the underlying mechanism for cognitive decline in individuals with SCD. Abnormalities in functional connectivity and node centrality measures of the DMN and frontal executive network are observed, leading to reduced brain network integration and increased segregation of functions (Xu et al., 2020). Subsequent studies have found that disruptions in the connectivity between the posterior DMN (pDMN) and the parahippocampal gyrus increase the risk of conversion from SCD to AD (Sharma et al., 2021). Enhanced functional connectivity in certain brain regions within networks serves as a compensatory mechanism for maintaining cognitive function in SCD. For instance, there is excessive connectivity between the right insula in LN and the hippocampus (Jiang et al., 2023); increased local or mid-range connectivity between the bilateral parahippocampal gyrus and other regions of the DMN (Chen H. et al., 2020); increased connectivity within the right FPN (Chen et al., 2021a); and hyperconnectivity within and between the visual, auditory, and sensorimotor networks (Chen et al., 2021b).

Furthermore, both the functional and structural integrity of the dorsal attention network (DAN) is compromised in SCD patients, with a significant decreased in functional connectivity between the left DAN modules. Research indicates that the connectivity of the DMN, ventral attention network, and sensorimotor network is similar between individuals with SCD and normal aging. However, the DAN shows greater specificity in predicting cognitive abilities (Jiang et al., 2022), making DAN a critical focus in diagnostic studies of SCD patients. There was a research that focused on the study of DAN brain region functional and structural imaging and used the ROI analysis method for detection. It was found that the fALFF ofthe left inferior parietal lobule was decreased, and this phenomenon can be regarded as an imaging index for differentiating SCD and MCI patients from the neurotypical individuals (Wu et al., 2023).

Patients with SCD have a decline in visual attention and short-term memory function, accompanied by complaints about their own attention, and also show symptoms such as anxiety and depression. There is a study found that the efficiency and centrality of the brain functional networks in SCD, as well as the volume of the hippocampus/parahippocampal gyrus, are preserved. This allows cognitive functions such as memory, language, and executive function to remain in a normal state, while levels of depression and anxiety show an increasing trend (Ren et al., 2022). This research corroborates the existence of an association between SCD and psychological aspects. However, the causal nexus between anxiety, depression, and SCD remains equivocal, and it is arduous to classify them as either cardinal symptoms or concomitant manifestations of SCD. A prior study hypothesized that anxiety and depression mediate the connection between sleep disorders and SCD symptoms, positing them as etiological factors in SCD patients (Xu et al., 2021). Despite an ample sample size, the cross-sectional nature of the study imposed constraints on causal inference. A 2017 study also identified anxiety and depression as influencers of the intensity of self-reported cognitive complaints in SCD (Tandetnik et al., 2017). To sum up, the elevated prevalence of depression and anxiety among SCD patients is well-established. Presently, a preponderance of studies lean toward considering anxiety and depression as risk factors for the progression toward SCD.

Declines in visual attention and short-term memory function are evident in the early stages of the AD continuum. A study on disruptions in the parieto-occipital network associated with visual attention and short-term memory found that the SCD stage exhibited network characteristics (clustering coefficient, strength, betweenness centrality) similar to MCI (Lazarou et al., 2022). This finding confirms the evolutionary trajectory of the AD continuum, although the study was conducted using electroencephalography neurophysiology, differing from the imaging topics addressed in this review.

Another common symptom of SCD is self-reported complaints about attention, which are significantly associated with reduced FC in the cingulo-opercular network. However, one study found a significant positive correlation between the forceps minor neurite density and the FC of the cingulo-opercular network (Ruiz-Rizzo et al., 2022). This suggests that structural damage to brain networks may contribute to further functional deterioration.

This indicates that the research results from different teams vary, possibly due to the heterogeneity of individuals with SCD, which leads to different manifestations of clinical symptoms. Overall, the clinical symptoms of SCD are not pronounced. However, at the aMCI stage, the functional abnormalities of FC, functional connectivity strength or functional connectivity density in regions, such as the DMN (Miao et al., 2022), Olfactory-Related Regions (Chen B. et al., 2022), cortical-cerebellar (Tang et al., 2021), and basal ganglia (Xiong et al., 2022). This indicates that by the MCI stage, abnormalities in functions such as visuospatial skills, language, and olfaction gradually emerge.

### 2.5 Metabolic abnormalities to the brain network

Research exploring the brain metabolic status of the AD continuum based on connectomics utilizes Aβ-positron emission tomography (Aβ-PET), tau-PET, 18F-fluorodeoxyglucose PET (18F-FDG PET), and arterial spin labeling (ASL) for scanning. These techniques detect the deposition of Aβ plaques, tau distribution, glucose metabolism, and cerebral blood flow (CBF) within brain networks.

It has been reported that amyloid proteins can be detected by PET scans in 25–30% of cognitively neurotypical older. Further studies have found that the rate of brain structure atrophy in amyloid - positive patients is more susceptible to risk factors. They have a smaller hippocampus, higher axial diffusion of white matter tract properties (Molinuevo et al., 2014), differences in brain region activation levels, a more significant reduction in the functional connectivity of the DMN, and increased metabolism in specific brain regions. Thus, abnormal amyloid deposition is the pathological basis of disease progression and a specific characteristic of SCD (Janssen et al., 2022). However, earlier studies held the view that amyloid could not support the claim that it is more likely to progress to AD because it was found that the cognitive function of amyloid - positive individuals could remain normal (Aizenstein et al., 2008). This study was conducted with a small sample size, so it is not very persuasive but has sparked more debate. Notably, based on the experimental results, subsequent researchers speculate that abnormal hippocampal function activation may be a key biomarker of cognitive decline in SCD, and amyloid is a result of metabolic disorders due to aging (Chen X. et al., 2021).

All in all, due to the differences in research methodologies and samples adopted, researchers have formed diverse viewpoints. In the AD continuum, metabolic abnormalities are not limited to a single brain region. Researchers have keenly grasped this key point and thus led the academic community to move forward in the direction of exploring the overall metabolic situation of the brain network, expect to achieve more systematic and comprehensive breakthroughs in the research of this field.

Research has found a close association between self-reported physical weakness (Lim et al., 2023), decreased self-reflection (Demnitz-King et al., 2022), and decreased brain metabolism in the SCD stages. Based on the A/T (N) framework (Peretti et al., 2023), evaluating the association between the ATN spectrum—comprising biomarkers for amyloid (A), tau (T), and neurodegeneration (N)—and cognitive decline. It has been observed that the Aβ structural connectivity is negatively correlated with local structural connectivity (Yu et al., 2021). However, the Aβ functional connectivity mainly gathered in the central hub of the DMN (Fountain-Zaragoza et al., 2023), leading to a decline in intra-and inter-network connections within the DMN, expanding to the entire brain network as the disease progresses.

The accumulation and propagation of tau protein are often determined by brain structural and functional connectivity, while Aβ also plays a promoting role in tau deposition (Lamontagne-Kam et al., 2023). Tau protein initially accumulates in the medial temporal region, basal region, lateral temporal lobe, and posterior cingulate gyrus, ultimately spreading to the old cortical areas (Bitra et al., 2023; Cho et al., 2016). When it progresses to the AD stage, the widespread diffusion of tau is associated with cortical thinning in specific brain regions (Chen X. et al., 2023), further validating the relationship between tau protein and human brain structural connectivity. It is noteworthy that several studies have identified tau-PET as a promising imaging tool for predicting cognitive decline, demonstrating its superiority over Aβ-PET and MRI (Smith et al., 2023; Ossenkoppele et al., 2021).

SCD is the first stage in the AD continuum from being asymptomatic to developing AD. Research has found that self-reported physical frailty (Lim et al., 2023), decreased self-reflection (Demnitz-King et al., 2022) in individuals with SCD are closely associated with decreased brain metabolism. In studies of glucose metabolism networks in SCD, differences were found compared to neurotypical individuals. The SCD metabolic network exhibited a more regular structure, with higher clustering coefficients and local efficiency (Dong et al., 2020). Additionally, SCD patients carrying the APOE gene showed stronger metabolic connectivity than non-carriers, providing more practical evidence for the pathological mechanisms of SCD (Zhang Q. et al., 2023). In contrast, another study identified reduced FDG metabolism in the right middle temporal gyrus as a potential biomarker for SCD, as it was found that only the FDG levels in this region correlated with chief complaint symptoms (Dong et al., 2021). The FDG-PET technique used in these studies is highly sensitive in detecting neuronal activity changes caused by neurodegenerative diseases and serves as a primary tool for clinically assessing dementia, including AD-related dementia (Minoshima et al., 2022). This provides a theoretical basis for examining the metabolic network in SCD.

Furthermore, many researchers found that reduced CBF in specific brain regions by ASL sequence, particularly in the medial temporal lobe (Kapasouri et al., 2022). In this stage, signs of reduced cerebral perfusion appear in regions such as the hippocampal head, posterior cingulate cortex, middle temporal gyrus, inferior frontal gyrus, and fusiform gyrus (Yang et al., 2021; Hays et al., 2018), with more pronounced effects in the MCI stage. The abnormalities in CBF across various brain regions, including the precuneus, inferior parietal lobule, superior occipital gyrus, middle occipital gyrus, cingulate gyrus, superior temporal gyrus, and middle temporal gyrus during MCI stage (Tang et al., 2022; Camargo et al., 2021). Even more there is a positive correlation between CBF in the cingulate gyrus and volume atrophy, while CBF in the right superior temporal gyrus exhibits a negative correlation with volume atrophy. This indicates that areas of reduced brain perfusion in the MCI stage are more widespread and are coupled with neurodegenerative changes. This underscores the CBF to serve as a potential vascular biomarker for early AD diagnosis (Agnollitto et al., 2023; Niu et al., 2023). Although this conclusion was obtained through the research on the correlation between CBF and brain volume in MCI, AD and healthy populations, without the participation of SCD population, it is still of great value for the early diagnosis of SCD.

### 2.6 Treatment mechanism of SCD

Currently, there is a growing emphasis on the treatment of SCD patients, with pharmacological treatments not being the first choice. Instead, educational programs and physical interventions are prioritized. Educational programs include cognitive training, physical exercise, computer training, enhanced cognitive reserve, and psychosocial interventions, which provide protective effects and reduce the risk of SCD (Liou et al., 2021; Gong et al., 2024). Physical interventions involve various measures, with repetitive transcranial magnetic stimulation (rTMS) emerging as a notable treatment method that has shown significant effects. For example, rTMS is a treatment method that uses electrical and magnetic stimulation to influence neuronal activity in the brain. By stimulating different brain regions, researchers can observe the effects on the functioning of various areas and understand the changes in functional connectivity following the intervention. It was found that rTMS improves episodic memory in SCD patients by modulating the functional connectivity of the precuneus-hippocampal circuit (Chen J. et al., 2020). It also enhances cognitive function by adjusting the abnormal effective connectivity patterns within the DMN and the cognitive control network (CEN; Liang et al., 2024).

In summary, existing research suggests that educational programs are considered the preferred intervention for SCD, while the more complex mechanisms of rTMS require further investigation. In fact, studies on rTMS interventions are more prevalent in the MCI and AD stages. For instance, after rTMS intervention, assessing whether the intrinsic functional connectivity within specific networks, such as the DMN and FPN, as well as the left dorsolateral prefrontal cortex, has increased can help evaluate whether memory impairment in MCI patients has improved (Sharbafshaaer et al., 2023; Wang T. et al., 2023). Similarly, using the same approach, rTMS stimulation of the left dorsolateral prefrontal cortex and lateral temporal lobe can effectively activate the effective connectivity of cognition-related brain networks (LN, DMN), reshaping brain functional networks and subsequently improving cognitive performance in AD patients (Qin et al., 2023).

## 3 Future directions

Firstly, based on current research, multimodal analysis methods incorporating machine learning (ML), deep learning (DL), and artificial intelligence (AI) play a crucial role in uncovering specific spatial distribution information within the brain, identifying potential neuroimaging biomarkers across the AD spectrum, and elucidating the underlying brain network mechanisms associated with AD-related cognitive impairments, even contributing the diagnosis and conversion prediction of SCD (Zhang Y. et al., 2023; Mora-Rubio et al., 2023). By contrast, if only the brain morphology and white matter are evaluated, the results will not only be highly subjective but also have limited information. However, by leveraging various deep learning frameworks and combining the characteristics of global anatomical structures with white matter changes for differential diagnosis and predicting the AD continuum, the objectivity and reliability can be significantly improved (Gao X. et al., 2023; Hoang et al., 2023). Meanwhile, studies based on models such as ML, DL and AI on the brain functional connectivity and metabolomics of the AD spectrum have provided scientific imaging evidence for the early diagnosis of SCD (AlSharabi et al., 2023; Kim et al., 2023; Jo et al., 2023; Duan et al., 2023). For example, studies have found that the volume of perivascular spaces (PVS) in the centrum semiovale may be a very early imaging biomarker for AD, and the impairment of perivascular clearance ability, which is part of brain metabolomics, is regarded as a risk factor for AD. Whether there are changes in the PVS among individuals with SCD still requires further research. This also provides a relatively innovative direction for the early diagnosis of SCD. Overall, data mining means such as machine learning, deep learning and artificial intelligence have many advantages in the clinical application of the AD continuum, but they also face challenges. For example, the application models require a large amount of labeled data for training, are prone to overfitting, have relatively high requirements for computing resources, have poor model interpretability, are relatively sensitive to label noise and data deviation, and are difficult to adjust and optimize parameters. In order to build more comprehensive models to predict the development of the AD continuum, it is necessary to conduct in-depth exploration and improvement in aspects such as improving data quality, optimizing algorithm design and enhancing model interpretability, so as to promote the more accurate and effective application of these technologies in the field of AD diagnosis.

Secondly, the investigation into multimodal brain network analysis in the continuum of AD remains an area in need of further exploration. Presently, the potential interconnections among functional, structural networks, and other aspects of brain network research lack clear definition. Multiple studies have illustrated that multimodal imaging analysis yields higher Area Under the Curve (AUC) values in Receiver Operating Characteristic (ROC) curves, indicating superior classification quality compared to single-modal analyses (Zhang J. et al., 2023; Chand et al., 2022). Studies have found that multimodal brain networks improve the detection accuracy of SCD (Chen H. et al., 2022; Lei et al., 2021), which provides a successful case for the brain network research of SCD patients. For example, the integrated analysis of gray matter and white matter can provide a more comprehensive understanding of the correlations among brain tissues and plays a crucial role in the brain effect mechanism of SCD (Liang et al., 2021). In summary, the pursuit of multimodal fusion analysis of brain networks represents a direction for future neuroimaging research in SCD, aiming to address, resolve, and explore various challenges. Despite the complexities associated with understanding the principles and mechanisms of each technology, the inherent drawbacks of different techniques, and the risk of model overfitting or underfitting, this approach shows promise in advancing our comprehension of the SCD.

Finally, a practical issue remains to be addressed promptly: how to aggregate the current and prospective research achievements in this domain, thereby formulating a unified diagnostic criterion and implementing it in clinical practice. This constitutes the core task for future investigators to explore intensively. Essentially, it is incumbent upon scholars to comprehensively review, precisely annotate, validate via literature search, and accurately demarcate relevant standards of the extant research outcomes. Primarily, a comprehensive evaluation framework is to be constructed, integrating neuroimaging biomarkers and cognitive assessments, and devising diagnostic benchmarks and clinical application protocols. Subsequently, personalized and lifestyle interventions are to be carried out. Eventually, a monitoring and follow-up regime, such as digital surveillance and multi-center clinical research consortia, is to be established. Paramount among these is the establishment of a comprehensive evaluation framework, which also represents the research objective of this review and becomes a crucial step in facilitating the clinical application of SCD research findings.

## 4 Conclusion

In the above explanation, it is concluded that the brain network neuroimaging research provides a more reliable means to distinguish healthy people, SCD, MCI and AD. At the same time, it is also a powerful medicine to deeply strengthen the goals and purposes of the brain science development plan “understanding the brain,” “protecting the brain” and “creating the brain,” laying the foundation for the research on the brain working mechanism. How to accurately diagnose SCD in the population is the most concerned problem at present. Various research results show that early diagnosis of SCD and early preventive measures can not only reduce the incidence of AD disease spectrum, but also improve the quality of life of patients, even integrating numerous scientific research achievements into unified and established standards and applying them in clinical practice by doctors, all prompting further research by researchers to provide solutions to the problem of SCD in the older adults.
